# Engineered Bacteria-Based Living Materials for Biotherapeutic Applications

**DOI:** 10.3389/fbioe.2022.870675

**Published:** 2022-04-28

**Authors:** Rabia Omer, Muhammad Zubair Mohsin, Ali Mohsin, Bilal Sajid Mushtaq, Xumeng Huang, Meijin Guo, Yingping Zhuang, Jiaofang Huang

**Affiliations:** ^1^ State Key Laboratory of Bioreactor Engineering, East China University of Science and Technology, Shanghai, China; ^2^ State Key Laboratory of Food Science and Technology, School of Food Science and Technology, National Engineering Research Center for Functional Food, Jiangnan University, Wuxi, China

**Keywords:** engineered living materials, synthetic live therapy, synthetic biology, biodiagnostic, biotherapeutics, multiplex diseases

## Abstract

Future advances in therapeutics demand the development of dynamic and intelligent living materials. The past static monofunctional materials shall be unable to meet the requirements of future medical development. Also, the demand for precision medicine has increased with the progressively developing human society. Therefore, engineered living materials (ELMs) are vitally important for biotherapeutic applications. These ELMs can be cells, microbes, biofilms, and spores, representing a new platform for treating intractable diseases. Synthetic biology plays a crucial role in the engineering of these living entities. Hence, in this review, the role of synthetic biology in designing and creating genetically engineered novel living materials, particularly bacteria, has been briefly summarized for diagnostic and targeted delivery. The main focus is to provide knowledge about the recent advances in engineered bacterial-based therapies, especially in the treatment of cancer, inflammatory bowel diseases, and infection. Microorganisms, particularly probiotics, have been engineered for synthetic living therapies. Furthermore, these programmable bacteria are designed to sense input signals and respond to disease-changing environments with multipronged therapeutic outputs. These ELMs will open a new path for the synthesis of regenerative medicines as they release therapeutics that provide *in situ* drug delivery with lower systemic effects. In last, the challenges being faced in this field and the future directions requiring breakthroughs have been discussed. Conclusively, the intent is to present the recent advances in research and biomedical applications of engineered bacteria-based therapies during the last 5 years, as a novel treatment for uncontrollable diseases.

## Graphical Abstract

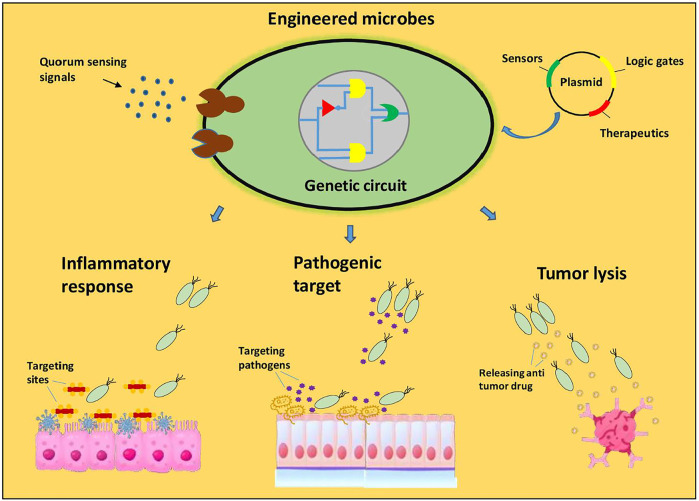

## Introduction

The development of dynamic and intelligent living materials is required to predict and diagnose illness as systemic drug administration cannot meet the demand of precision medicine. Additionally, systemic drug administration is associated with toxic side effects as it provides off-target delivery with a high number of doses in unwanted locations (Ozdemir et al., 2018). On the contrary, advances in biotechnology enable the use of natural living materials, including cells, microbes, biofilms, and spores, for targeted delivery and remote activation (Mohsin et al., 2021; Tang et al., 2021).

**FIGURE 1:**
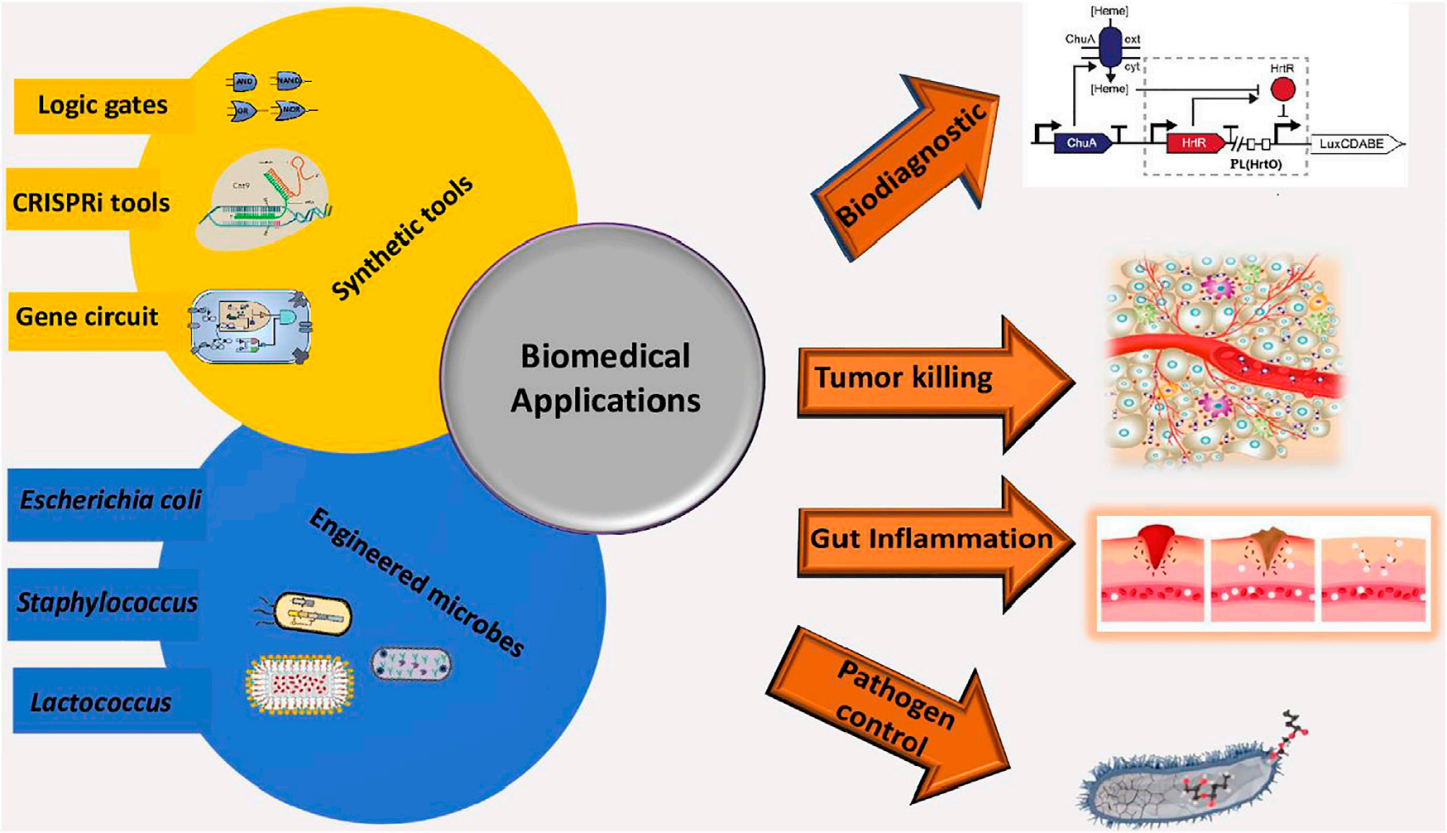
Graphical illustration of genetically engineered microbes by using synthetic biology tools. These engineered bacteria-based living materials are used for biodiagnostics and therapeutic purposes.

The concept of engineered living materials (ELMs) is an emerging class that consists of engineered living entities capable of modifying the characteristics of the material itself (Nguyen et al., 2018; Guo et al., 2020). In this regard, synthetic biology contributes to clinical applications by engineering living entities, as shown in Figure 1.

It allows the reprogramming of microbes by providing well-characterized genetic parts and circuits (Gilbert and Ellis, 2019; Burgos-Morales et al., 2021). Moreover, sophisticated genetic circuits have been developed with the help of molecular tools to perform complex tasks, gaining attention day by day (Barra et al., 2020b). These living materials can be reprogrammed as sensing machines to detect external stimuli and respond to various cellular tasks. In ELMs, the biological entities impart living properties to materials, such as bio-sensing, self-regeneration, and computational capabilities at the molecular level (Huang et al., 2019; Pu et al., 2020). At the same time, material encapsulation provides applications, such as protection, mechanical enhancement, and communication with engineered living entities (Heveran et al., 2020; Caro-Astorga et al., 2021). Consequently, remarkable attributes can be attained, such as high specificity and sensitivity to poor stimuli, better strength, and the ability to sense stimuli continuously (Inda and Lu, 2020).

Microorganisms, particularly probiotic bacteria such as *Lactobacillus* spp. and *Bifidobacterium* have been engineered for synthetic living therapies. Also, *Bacillus* spp., *Streptococcus*, and *Escherichia coli* are among the bacteria identified for therapeutic interventions because of the detailed characterization of the human microbiota (O’Toole et al., 2017). These programmed bacteria provide targeted delivery of anticancerous and antimicrobial enzymes to treat various cancers and infections, respectively. On the contrary, chemotherapeutic agents are toxic, disrupting the normal cell cycle to slow down the proliferation of abnormal cancer cells and affecting the cycle of healthy cells (Pearce et al., 2017). Thereupon a huge interest is present in developing solutions for targeted cancer therapy. A 3D model of diseases can be an excellent platform for quantifying bacterial localization and circuit dynamics, which is essential for accurate *in vivo* safety and effectiveness in bacteria-based treatments. *Staphylococcus typhimurium* delivers a variety of anticancer compounds through synthetic genetic manipulation, which makes it effective for *in vivo* treatments (Harimoto et al., 2019). Meanwhile, biotherapeutics with targeted administration and activity in the gut could also be a promising alternative to meet current requirements for inflammatory bowel disorder treatment (Hazel and O’Connor, 2020).

The main purpose of writing this review is to provide insights into the biomedical applications of engineered bacteria for the treatment of multiple diseases. Such synthetic microbes are the best option because of their adaptive and metabolic behaviour and can relieve humankind from the curse of diseases. Although this field is growing rapidly and has generated substantial interest in designing new and novel materials, some challenges still need to be addressed. Finally, we will discuss the challenges being faced in this field and the future directions requiring breakthroughs.

## Role of Synthetic Biology in Engineering Microbes

Synthetic biology provides a platform for reprogramming the biological functions of living components to obtain specific and desired responses. It is the most common route to design bacteria to express proteins that can produce quantifiable signals in response to target stimuli (Martin-Yken, 2020). By using genetic circuits, the expressive genes of the microbiome can be controlled in a spatiotemporal manner. Genetic circuits are the assembly of genetic parts and regulatory modules that are used to fine-tune the therapeutic functions of microbes (Riglar and Silver, 2018). Synthetic biology allows the construction of synthetic circuits to harness these regulatory mechanisms, which is an effective approach to treat and target drug release. The introduction of synthetic circuits controls the expression of transcription factors (TFs) and allows the design and construction of new synthetic networks for next-generation biomedical applications ranging from sensing toxic compounds to probing and eliminating pathogens and delivering useful compounds to targeted sites (Rondon et al., 2019).

**FIGURE 2:**
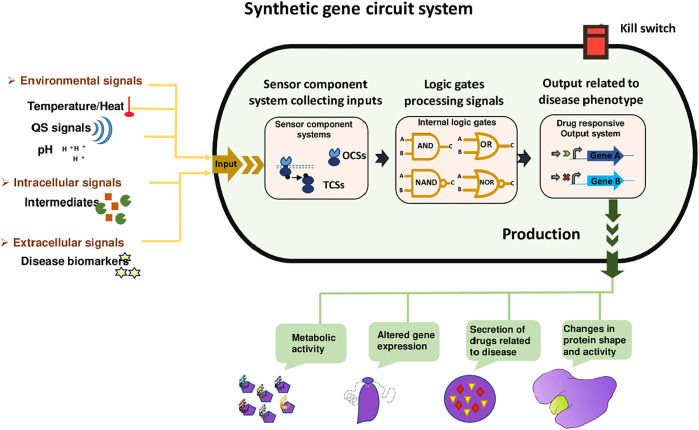
Synthetic gene Circuit System. It has three sections, including a sensing unit that can sense environmental signals (temperature, heat, and pH), intracellular signals (intermediates), and extracellular signals (disease biomarkers). These signals are received by the signal processing unit that processes the signals with the combination of logic gates to actively sense target stimuli. Moreover, AND, NAND, OR, and NOR are complex logic gates employed for multilayered logical processing. Finally, the actuator dictates the output of system parameters, for example, the modulation of gene expression and enzyme proteolysis ultimately hikes desired biological functions and phenotypes, such as cell growth regulation, cell morphology, fate, and motility change. Kill switches are usually used in designed bacteria so that their population can be controlled after accomplishing their task.

The synthetic circuit system as shown in Figure 2 consists of 1) Sensors; including riboswitches, TFs, and one and two-component systems. These sensors receive signals from the external environment, including light, temperature, pH, also respond towards extracellular signals, including biomarkers, quorum sensing (QS), and intracellular signals like intermediate metabolites and the cellular physiological state. Hundreds of genetically encoded sensors have been built using a one-component system (OCS) and a two-component system (TCS), to diagnose diseases. OCS comprises cytoplasmic TFs, which interact directly and are regulated by the bacteria-based physiochemical signal transduction system that transfers stimuli to the cells (Landry and Tabor, 2017). 2) Signal processing unit; that processes the signals with the combination of logic gates to actively sense multiple target stimuli. Moreover, genetic amplifiers allow the programmed recognition of various inputs as well as amplify the expression of the desired outcome. AND, NAND, OR, and NOR are complex logic gates employed for multilayered logical processing e.g., amplifiers, oscillators, toggle switches, and feedback/forward loops (Jo et al., 2021). 3) Output subsystem; the actuator dictates the output of system parameters according to the upstream processing subsystem. For example, the modulation of gene expression and enzyme proteolysis ultimately hikes desired biological functions and phenotypes, such as cell growth regulation, cell morphology, fate, and motility change (Moraskie et al., 2021).

Moreover, synthetic biology, combined with CRISPR (clustered regularly interspaced short palindromic repeats), is used for drug target screening. CRISPR is a highly programmable technology that proposes greater and more innovative features than classic tools for constructing synthetic gene circuits (Santos-Moreno and Schaerli, 2020). The construction of intelligent and genetically synthesized circuits based on CRISPR/Cas systems, such as artificial switch-inducible Cas9, provides better and targeted therapy of tumor cells over traditional single-targeted therapies unable to distinguish tumor cells from normal cells and are less efficient (Zhou et al., 2019). Scientists have used clustered regularly interspaced short palindromic repeats interference (CRISPRi) to construct a synthetic oscillator (“CRISPRlator”), a bistable system, and a stripe pattern-forming incoherent feedforward and backward loop in a population of *E. coli* to perform vigorous circuit behaviors (Santos-Moreno et al., 2020). CRISPR is commonly applied for the discovery of antibiotic-resistant genes. Furthermore, the CRISPR-Cas system combined with Combinatorial Genetics En Masse (CombiGEM) facilitates the systematic analysis of high-order genetic perturbations necessary to discover therapeutic target combinations and understand biological activities (Zhou et al., 2021).

The genetically engineered microbes should be equipped with a containment system if administered to humans. This system controls the bacterial population and prevents recombinant DNA from spreading into the environment. For this purpose, kill switches are used that are highly stable under permissive conditions while showing lethal activity in non-permissive conditions (Riglar et al., 2017:; Ceroni et al., 2018).

## Engineered Bacteria for Biodiagnostic Applications

**FIGURE 3:**
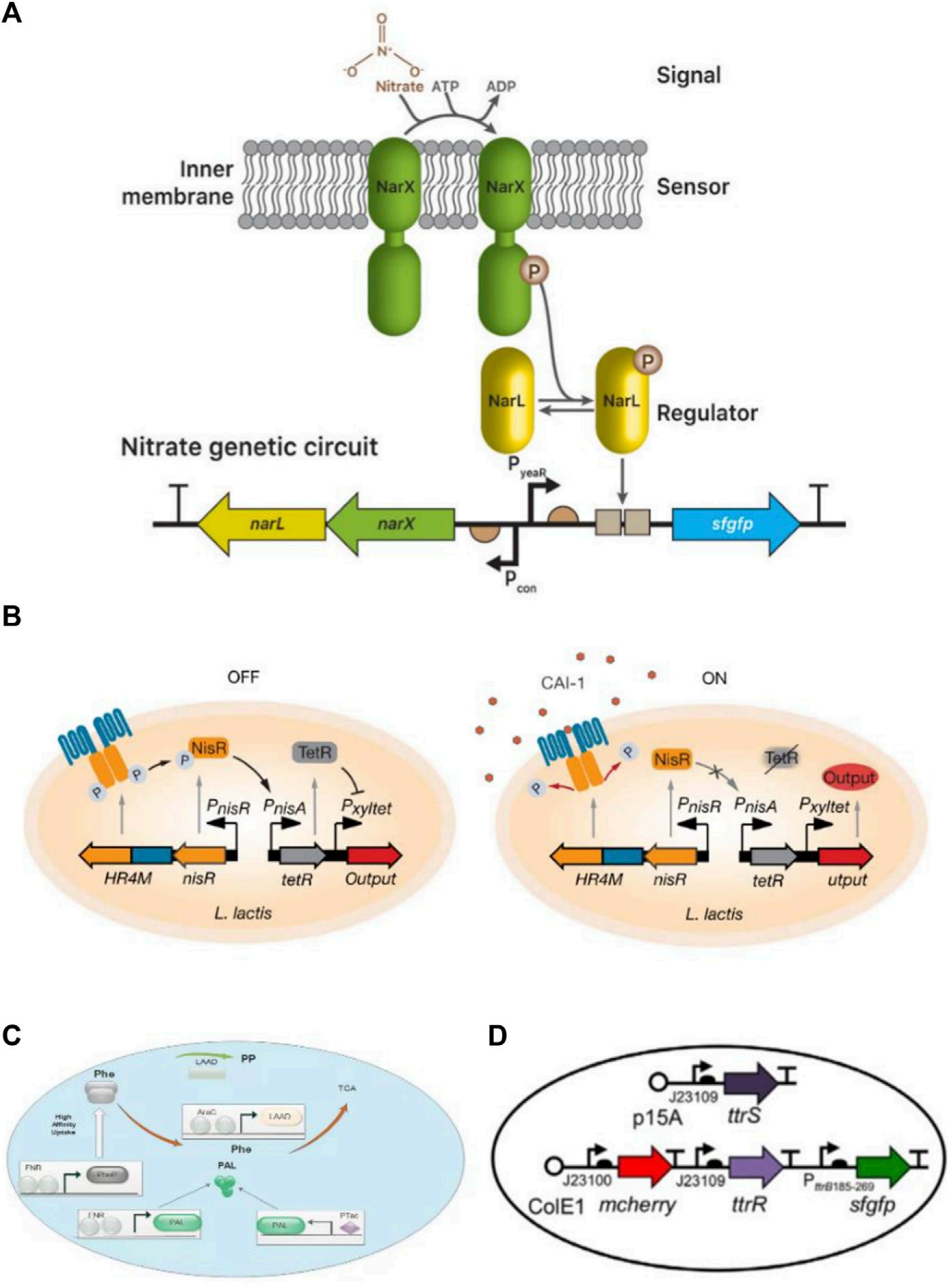
Biodiagnostic applications of engineered bacteria. **(A)** A nitrate sensing genetic circuit consists of a two-component system NarX and NarL. In nitrate presence, both become phosphorylated and drive the expression of superfolder green fluorescence proteins (sfgfp) reporter through a promoter. Reproduced with permission from Woo et al. (2020), Copyright 2020, Elsevier. **(B)** Vibrio cholera sensing device based on sensing module HR4M-NisR and processing module TetR/Pxyltet make a complete circuit. In the presence of CAI-1, phosphorelay prevents HR4M-NisR and reduces TetR expression generate output by the activation of the gene. Reproduced with permission from Mao et al. (2018), Copyright 2018, American Association for the Advancement of Science. **(C)** A therapeutic strain SYNB1618 consists of a gene for PheP encoding. PheP shows a high affinity towards Phe transporter that transports Phe into the cell and converts into TCA. **(D)** Plasmid design construction for the constitutive sensor in E. coli Nissle. Reproduced with permission from Daeffler et al. (2017), Copyright 2017, John Wiley and Sons.

Synthetic biology is focusing on the genetic engineering of bacteria to detect the states of diseases and intelligently deal with various diseases. In recent years diagnostic and therapeutic systems have been built by rewiring the signaling pathways of natural systems, as shown in Figure 3. Also, the list of promoters that are used in engineering microbes as sensing part has been summarized in Table 1. These designed bacteria are used as biosensors to detect the states of diseases.

**TABLE 1:** List of promoters used in biosensors.

Engineered strain	Promoters	Reporters	Applications	References
*E. coli* NGF-1	TtrSR-PttBCA	β-galactosidase	Tetrathionate responsive circuit diagnoses gut inflammation	Riglar et al. (2017)
*E. coli* Nissle	ThsSR- Pphsa	Green Fluorescence Protein (GFP)	Thiosulphate sensitive circuit senses gut inflammation	Daeffler et al. (2017)
*E. coli* NGF-1	TetR-Ptet	β-galactosidase	aTc sensitive detects cell division	Ceroni et al. (2018)
*E. coli* Nissle	HrtR-PL (HrtO)	luxCDABE	Heme sensitive circuit responds towards gut bleeding	Mimee et al. (2018)
*L. lactis*	Engineered Chimera	β-lactamase	CAI-1 responsive circuit detects cholera infection	Mao et al. (2018)
*E. coli* Nissle	TtrSR-PttrB	GFP	Tetrathionate responsive circuit detects gut inflammation	Daeffler et al. (2017)
*E. coli* Nissle	NarXL-Pyear	Superfolder green fluorescent protein (sfgfp)	Nitrates responsive circuit senses gut inflammation	Woo et al. (2020)

The simplest biosensor is nitric oxide (NO). Heme-NO/oxygen binding (H-NOX) domains are present in primary NO sensors that can identify nanomolar NO and influence downstream signal transduction in various bacterial species. Another primary NO sensor that uses bacterial hemoprotein is the NO sensing protein (NosP). NosP is a histidine kinase signal transduction pathway regulator and is responsible for biofilm formation in *Pseudomonas aeruginosa.* It can also be used as a substitute for H-NOX. To check its activity, *NorR* was inserted into the DNA switch with FimE-DNA recombinase, and a reversely oriented upstream promoter. Hence, it was noted FimE activated by *NorR* induced DNA inversion on the reversely oriented promoter of the reporter into the correct orientation and confirmed the NO presence by fluorescence signals (Williams et al., 2018). In another study, Woo et al. (2020) built a nitrate responsive genetic circuit by using a two-component system (NarX-NarL) in *E. coli* that can respond to the high level of nitrate during gut inflammation. Moreover, the genetically encoded sensor was developed by using Boolean AND gate for simultaneous monitoring of thiosulfate and nitrate gut markers (Woo et al., 2020).

Additionally, a commensal murine *E. coli* strain has been engineered that can sense tetrathionate, an inflammation biomarker. It shows memory in the presence of tetrathionate which drives Cro and β-galactosidase (β-gal) expression from the synthetic memory element. The engineered strain retained memory for up to 6 months and significantly affected diagnostic gastrointestinal inflammation (Riglar et al., 2017). Similarly, Daeffler et al. (2017) developed a system that can detect inflammation in the colon. This tetrathionate sensor device is based on a TCS, ttrS/R. Both studies confirmed the status of bacteria as live biodiagnostics (Daeffler et al., 2017). In another study, *E. coli* was designed to show heme sensitivity, capable of precise analysis of gastrointestinal leakage or bleeding. This heme-sensing gene circuit consists of the transporter ChuA internalized by heme. Heme interacts with HtrR (transcriptional repressor) to activate the expression of the *luxCDABE* operon. Such an ingestible micro-bio-electronic device (IMBED) provides an opportunity to successfully monitor compounds *in situ* and efficiently handle gastrointestinal disorders (Mimee et al., 2018).

The developed system has the potential to identify biomarkers associated with a range of diseases and can be estimated by successful clinical trials. Consequently, bacterial cell-based biosensors also perform activities based on sense and response schemes. For example, detecting a biomarker for a disease can elicit a response by releasing the corresponding therapeutic agents. Therefore, these biosensors also help in the treatment of metabolic disorders, such as phenylketonuria, in which phenylalanine (Phe) is unable to degrade, resulting in neurotoxicity during accumulation. In an anoxic environment, the engineered *E. coli* Nissle (1917) has the potential to metabolize Phe by releasing enzymes in the gut. Continuous success is needed to gain regulatory approval. If granted then it would be the first synthetic biology-based clinical treatment (Isabella et al., 2018). Also, a receptor in *L. lactis* is genetically transformed to detect the cholera autoinducer-1(CAI-1) gene from *V. cholera,* which can be detected and controlled using *L. lactis*. Lactic acid counteracts the production of *L. lactis*, thereby significantly decreasing the intestinal *V. cholerae* load and increasing survival rates of infected baby mice (Mao et al., 2018). Genetic engineering of viable cells is becoming technically simpler; therefore, unique and novel techniques are still required to generate engineered microbes in proper matrices that help to create stimuli-responsive ELMs.

## Engineered Bacteria for Disease Management

The naturally occurring microbes associated with the human body open up numerous opportunities and breakthroughs in biotechnology, which enable these microorganisms to use *in situ* therapies. Mainly probiotics, such as *Lactobacillus* spp. and *Bifidobacterium,* are used to engineer various therapies. However, *Bacillus* spp., *Weissella* spp., and *E. coli* are also among the bacteria identified for therapeutic interventions because of the detailed characterization of the human microbiota (O’Toole et al., 2017). The recent applications of engineered strains are listed in Table 2. The delivery of therapeutic agents and *in vivo* production by bacterial strains are more efficacious than traditional treatments. Unlike conventional medications, living therapies may provide continuous treatment for chronic illnesses with constant propagation. They can also administer anticancer drugs *in situ*, avoiding the harmful effects of systemic therapy on healthy cells. Finally, living therapies may be used to combat the rising number of antibiotic-resistant diseases (Landry and Tabor, 2017). Over the years, molecular methods for designing microbial living therapies have become more diversified, allowing the creation of sophisticated genetic materials for the treatment of complicated biological disorders. Hence these engineered microbes are likely to be included in next-generation microbial treatments. The use of bacteria as live therapy is practiced wherein programmable bacteria produce drugs, enzymes, and immunotoxins to treat various diseases, including cancers, inflammatory bowel diseases, and infections.

**TABLE 2:** Microbial based therapy.

Engineered strain	Disease	Study	Therapeutic Substance	Applications	References
*Lactococcus lactis*	Type2 Diabetes	*In vitro*	rExd4	Enhances glucose-dependent insulin secretion and activates the PI3-K/AKT signal pathway	Zeng et al. (2017)
*Lactococcus lactis*	Mucositis	*In vitro*	Pancreatitis-associated protein	It reduces intestinal inflammation by preventing 5-fluoracil	Carvalho et al. (2017)
*Lactobacillus plantarum*	Colorectal cancer	*In vitro*	Gamma-aminobutyric acid	Activates anti-proliferative, anti-migration, and anti-invasion effects against 5-FU-resistant HT-29 cells	An et al. (2021)
*Lactococcus lactis*	Cancer	*In vivo*	Anti-tumor-genic human TRAIL	Capable of inducing effective elimination of human colon cancer cells *in vivo*	Ciaćma et al. (2018)
*Lactobacillus plantarum*	Colorectal cancer	*In vivo* and *In vitro*	YYC-3	It reduces colon cancer metastasis	Yue et al. (2020a)
*Lactococcus lactis*	Rheumatoid arthritis	*In vivo*	Hsp65-Lac	Prevent the induction of chronic and acute models of arthritis	Gusmao-Silva et al*.,* (2020)
*Lactococcus lactis*	*H. pylori* infection	*In vivo*	HpaA	It provides anti-*H. pylori* vaccination with potent immunogenicity	Zhang et al. (2017)
*Lactococcus lactis*	*H. pylori* infection	*In vivo*	napA	Produces and delivers the oral vaccine against *H. pylori*	Peng et al*.,* (2018)
*Lactococcus lactis*	*Vibrio* cholera infection	*In vivo*	b-lactamase	Hinder cholera progression and improve disease surveillance in populations at risk of cholera outbreaks	Mao et al. (2018)
*Bifidobacterium longum*	Breast cancer	*In vitro*	Trastuzumab scFv	Provide *in situ* delivery to kill cancer cells	Kikuchi et al. (2017)
*Bifidobacterium longum*	Irritable bowel syndrome	*In vitro* and *In vivo*	LL-37	Provide therapeutic effect against bacterial diarrhea by reducing the population of *E. coli* and *S. aureus*	Guo et al. (2017)
*Bifidobacterium longum*	Inflammatory bowel disease		MnSOD	Reduced DSS-induced ulcerative colitis in mice	Liu et al. (2018)
*E. coli Nissle* 1917	VRE infection	*In vivo*	Enterocin A, Enterocin B, and Hiracin JM79	Specifically targeted and kill *Enterococcus*, potent activity against both *Enterococcus faecium* and *Enterococcus faecali*	Geldart et al. (2018)
*E. coli Nissle*	IBD	*In vitro* and *in vivo*	TFF	Treat inflammation and help to rebuild the intestinal epithelium	Praveschotinunt et al. (2019)
*Salmonella typhimurium*	Melanoma Cancer	*In vivo*	Interferon-gamma (IFN-γ)	*S. typhimurium* and IFN-g showed therapeutic potential for the treatment	Yoon et al. (2017)

### Tumor Targeted Therapy

**FIGURE 4:**
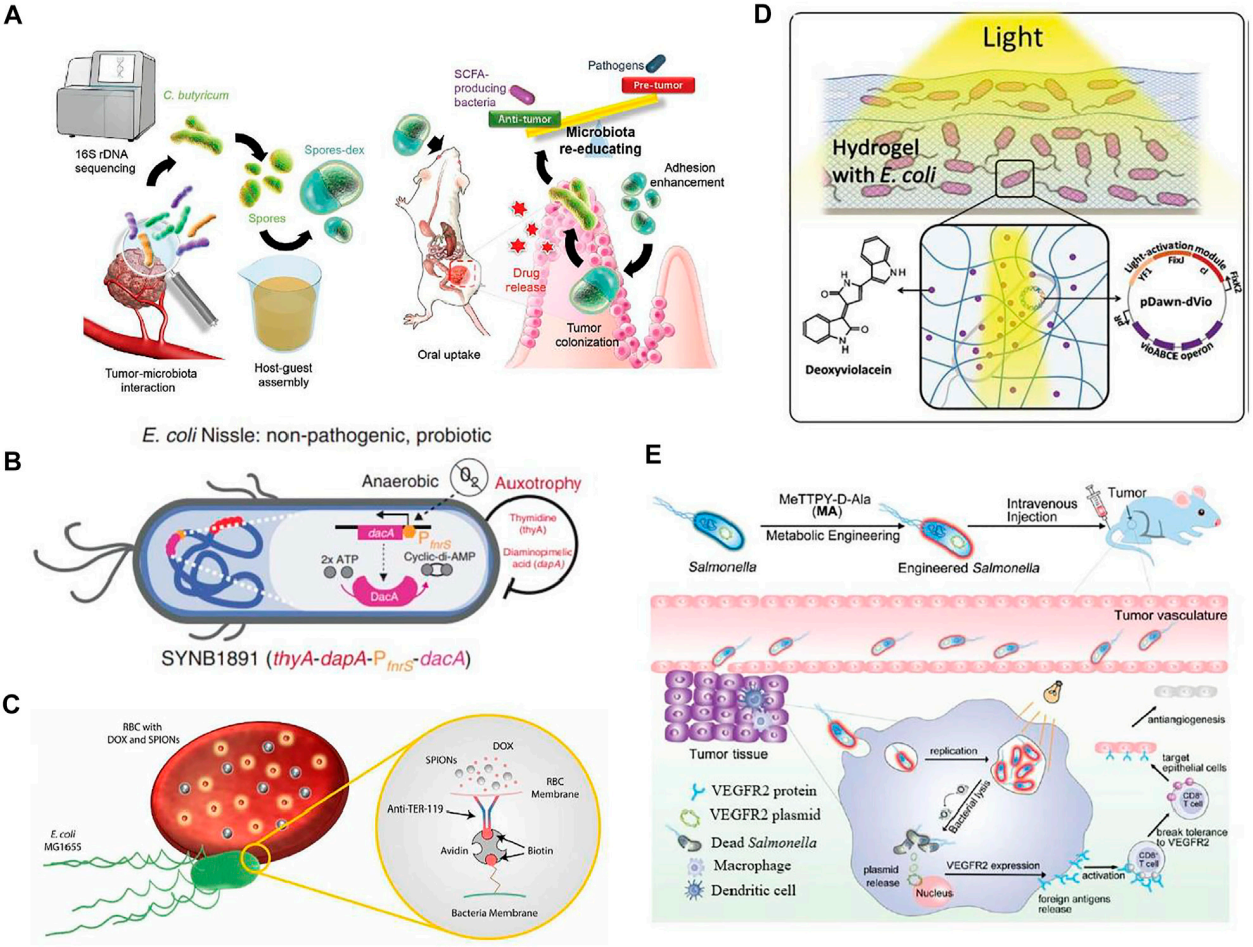
Applications of engineered bacteria for tumor therapy. **(A)** The drug (dextran)-loaded spores provide antitumor activity. Reproduced with permission from Zheng et al. (2020), Copyright 2020, John Wiley and Sons. **(B)** The engineered *E. coli* Nissle referred to as SYNB1891 consists of a biocontainment system having dapA and the auxotrophies, PfnrS dacA circuit integrated into the dacA gene promotes anaerobically production of CDA. This CDA expression promotes an anti-tumor activation mechanism. Reproduced with permission from Leventhal et al. (2020), Copyright 2020, Springer Nature. **(C)** The modified strainMG1655 consists of red blood cells loaded with doxorubicin (DOX) and superparamagnetic iron oxide nanoparticles (SPIONs) attached to modified motile *E. coli* bacterium *via* a complex, biotin-avidin-biotin. Reproduced with permission from Alapan et al. (2018), Copyright 2018, American Association for the Advancement of Science. **(D)** The engineered E.coli has pDawn-dVio, a blue-light responsive optogenetic plasmid that has the potential to produce drug deoxyviolacein in presence of light. Reproduced with permission from Sankaran et al. (2019), Copyright 2018, John Wiley and Sons. **(E)** Salmonella encodes the VEGFR2 plasmid engineered with MA and promotes anti-angiogenesis therapy of breast cancer *in vivo*. Reproduced with permission from Liu et al. (2021), Copyright 2021, Royal Society of Chemistry.

The rise of cancer leads to increasing concerns regarding the efficacy of currently available treatments. A therapeutic approach of using engineered bacteria may overcome the side effects of conventional cancer treatments. Scientists have continuously put their efforts into engineered bacteria having significant potential to target tumor sites and release therapeutic agents to kill cancer cells, as depicted in Figure 4. Considering this, *Salmonella* is most suitable for tumor therapy because of its ability to reside inside tumors and suppress their growth. However, it also has some limitations because it provides improper drug delivery, especially for bactofection delivery of therapeutic genes. With the help of metabolic engineering, *Salmonella* has been designed as a viable therapeutic system. The designed bacteria for controlled gene release therapy consist of vascular endothelial growth factor receptor 2 (VEGFR2) plasmids and the photosensitizer MeTTPy-D-Ala (MA). MA does not affect the growth of *Salmonella*; thus, it continues to grow in tumor tissues and releases genes in the presence of light irradiation. The VEGFR2 protein expression taken by host cells as a foreign antigen activates T-cells’ autoimmune response that targets epithelial cells. This facilitates tumor suppression by acting as an antiangiogenic (Liu et al., 2021).

Moreover, genetically modified bacteria are developed to produce myrosinase, an enzyme that converts dietary glucosinolate into the anticancer chemical sulphoraphane. This approach is beneficial as it inhibits >95% proliferation of colorectal cancer cell lines *in vitro* and suppresses tumor progression in an animal model (Ho et al., 2018).

Various bacteria have demonstrated considerable potential to colonize tumors selectively *in vivo*, thereby spurring efforts to design bacteria as programmable vehicles for administrating anticancer treatment. As a proof of concept, the engineered *Salmonella typhimurium* produces *Vibrio vulnificus* flagellin protein as a receptor-ligand, eliciting an immunogenic anticancer response in the host (Zhang et al., 2017). Recently, a combination of probiotic spores and prebiotic has been designed to consider the effective treatment of colon cancer. After oral administration, the prebiotic, dextran is fermented by *C. butyricum* and release anti-cancer short-chain fatty acids. While in subcutaneous and orthotopic tumors it has been observed that drug-loaded spores-dex inhibited tumor growth up to 89 and 65%, respectively (Zheng et al., 2020).

These biologically engineered bacteria offer a unique opportunity to develop a targeted and dynamic therapy that improves efficiency, accuracy, and safety compared to conventional chemotherapeutic treatments. This is because it is more effective in treating tumors as it used genetically engineered therapeutic payloads and control systems (Chien et al., 2017). Furthermore, bacteria exist in tumors, a new paradigm for cancer therapy. The researchers stated that the drug delivery system based on oncolytic bacteria has anticancer potential as they express anti-tumor molecules. These molecules provide direct tumor-killing nutrients secreted from dead cells, further promoting colonization. Localized oncolytic bacteria also regulate the immune system and promote the entry of macrophages and dendritic cells into a tumor to kill cells or inhibit their progression (Chen et al., 2021).

Another living biotherapeutic system has been developed by using *E. coli* Nissle as a dynamic platform for cancer treatment. The engineered strain is capable of targeting sting activation to phagocytic antigen-presenting cells (APCs) but also activates the innate immune system later. The treatment with engineered strain possesses antitumor immunity with immunological memory formation and vigorous activation of human APCs. This strain is built with biocontainment features so that its efficiency is not compromised. This work demonstrates the potential of synthetic biology in the development of drugs as living medicine, which shall strengthen the development of live biotherapeutics in the future (Leventhal et al., 2020).

In addition, targeted drug delivery can be achieved by modifying the characteristics of micro-swimmers and propulsion mechanisms. These swimmers have the potential to communicate with the outside environment and generate a response by computing the information as bacteria are naturally guided with the communication power (Sun et al., 2020). Hence, novel technological techniques such as microswimmers exploit the characteristics of bacteria to improve the quality of life. For example, Park et al. (2017) engineered multifunctional bacteria-driven microswimmers that exhibited high performance for drug delivery applications. These swimmers were *E. coli* bacteria incorporated with a drug loader, polyelectrolyte multilayer (PEM) microparticles embedded on magnetic nanoparticles (Fe_3_O_4_). They have significant potential to target specific cells following chemotactic and magnetic guiding. Further, the microswimmer was tested *in vitro* by loading doxorubicin, an anti-cancer drug on PEM. The swimmer under magnetic guidance can target the 4T1 breast cancerous cells. So, the results indicate that the use of active PEM microswimmers in drug delivery is feasible as it enhances drug transfer compared to passive PEM microparticles (Park et al., 2017).

Similarly, RBCs microswimmer was designed to transport cargoes into specific sites that consisted of red blood cells loaded with DOX and superparamagnetic iron oxide nanoparticles (SPIONs) attached to modified motile *E. coli* bacterium through a biotin-avidin-biotin complex. This complex provides robust attachment between bacteria and RBCs as well as stability in extreme conditions. These swimmers swim due to flagella of bacteria while guided by SPIONs. Hence RBCs are a promising candidate for targeted drug delivery as they provide efficient drug release. Moreover, their population is controlled by control switches that are activated by light (Alapan et al., 2018).

Recently, the optogenetically controlled living material has been designed that has the potential to sense light irradiations and respond by producing therapeutic proteins. Irradiation triggers-controlled drug release delivery drug on intended locations. The metabolically engineered *E. coli* can produce various drugs; most important are bisindoles dVio and violacein as they show antibacterial, antifungal, and antitumoral characteristics. An optogenetically engineered *E. coli* has been developed by the incorporation of the enzyme VioABCE operon within the pDawn, a light-responsive plasmid. This blue-light responsive plasmid can produce drug deoxyviolacein in the presence of light. It is encapsulated onto two different hydrogel matrices, one is covalently cross-linked polyacrylamide (PAAM), and another is physically cross-linked agarose. Both matrices have shown considerable functional drug production and deliver drug doses controlled by light. Hence, the engineered strain can produce and release the drug for 42 days, offering advanced therapeutic applications (Sankaran et al., 2019).

On the other hand, microrobots are envisioned for clinical applications as they supply drugs to specific spots. In a study, a magnetic microrobot was designed in which bacteria *Spirulina* (*Sp.*) was used as a scaffold material. The purpose was to load doxorubicin (DOX) and photothermal agents to increase therapeutic efficiency. *Sp*. cells loaded with Pd@Au nanoparticles and then Fe_3_O_4_ nanoparticles were deposited on the obtained surface (Pd@Au)@Sp by the sol-gel process exhibit movement in a magnetic field. Finally, DOX was loaded further on (Pd@Au)/Fe3O4@Sp. These robots exhibited the highest propulsion with 526.2 μm/s speed following a rotating magnetic field. Also, laser irradiation disassembles into individual parts and exhibit pH- and NIR-triggered drug release. These characteristics make them an efficient and promising platform for targeted delivery and drug loading (Wang et al., 2019). There is no doubt that the therapeutic approaches for cancer are significant, but the fight against cancer is not easy. Hence, more novel and creative therapies are required to harness the potential of bacteria to cure such diseases.

### Inflammatory Bowel Diseases (IBD’s)

**FIGURE 5:**
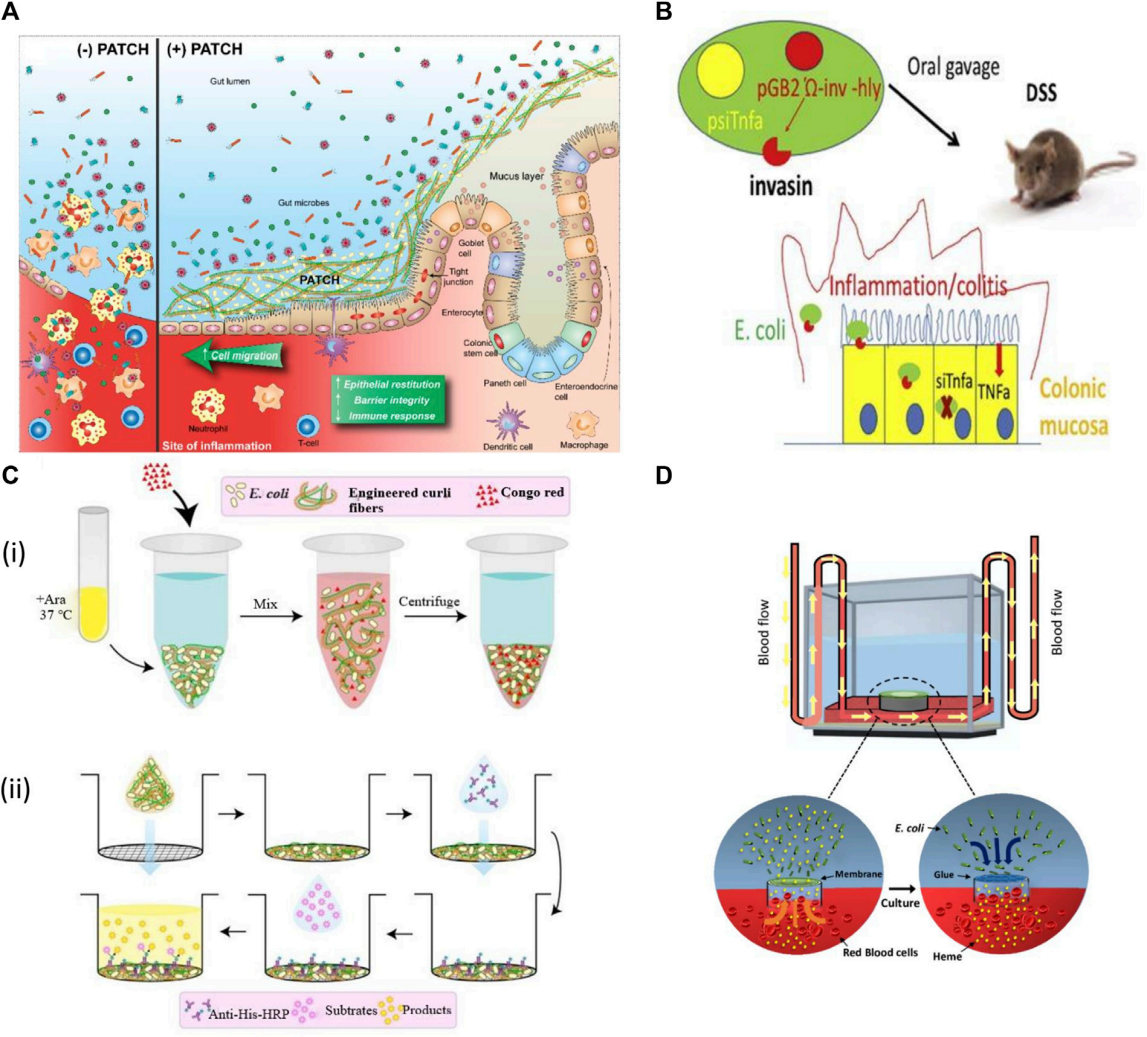
Applications of engineered bacteria in IBDs. **(A)** Probiotic Associated Therapeutic Culi Hybrids (PATCH) and colonic mucosa interaction. IBD causes inflammatory wounds that result in damaged epithelial tissue, loss of colonic structure with less barrier integrity (Patch−). The Patch application produces curli fibers result in enhanced intestinal barrier function, restitution of epithelial tissues, and lowers immune response to improve the IBD activity (Patch+). Reproduced with permission from Praveschotinunt et al. (2019), Copyright 2019, Springer Nature. **(B)** The modified E. coli MDS42 strain expresses invasion protein and listeriolysin O that helps to invade and damage phagosomal membrane respectively. This process allows the plasmid delivery encoding shRNA downregulates TNF. While the inflammation was also decreased in DSS-induced colitis. Reproduced with permission from Ferenczi et al. (2021), Copyright 2021, Elsevier. **(C)** (i) The curli fiber variants produced from engineered E. coli Nissle at 37°C in LB media containing arabinose (Ara) measured by Congo Red Binding Assay. (ii) Illustration of the whole-cell filtration ELISA for measuring the engineered curli fibers produced from bacterial culture. Reproduced with permission from Praveschotinunt et al. (2019), Copyright 2019, Springer Nature. **(D)** A living glue system enables repairing as it can sense heme coordination complexes and then respond by repairing the leakage sites within a microfluidic device channel.

Besides cancer, IBDs are also among the most common diseases. Ulcerative colitis (UC) and Crohn’s disease are inflammatory bowel diseases (IBDs). Despite receiving different therapies throughout their lifetimes, many IBD patients ultimately need surgery to address problems and relieve symptoms. Therefore, new treatment preferences should focus on improving intestinal epithelium integrity instead of merely treating symptoms. Recent developments in synthetic biology have increased the reliability and authenticity of bacterial designs with better genetic modification, biological designs, and actuator genes, which are required for diagnostic and therapeutic activities of inflammatory disease, as shown in Figure 5 (Landry and Tabor, 2017).

Live biotherapeutics with targeted administration and activity in the gut could be a promising alternative to meet current requirements for inflammatory bowel disorder treatment (Hazel and O’Connor, 2020). For example, *E. coli* Nissle (1917) has recently been engineered to produce an extracellular matrix of trefoil factors (TFFs) for inflammation and could reform the intestinal epithelium (Praveschotinunt et al., 2019). TFFs were fused to the C-terminus of CsgA; the CsgA-TFF fusion gene was transcribed then with other genes for the formation and secretion of curli fibers. This synthetic curli operon was controlled by an arabinose-induced promoter, incorporated into the plasmid pBbB8k with a kanamycin selection marker. When this engineered strain was delivered orally to mice with dextran sodium sulphate (DSS) induced colitis, it was confirmed that it could produce curli fibers with protective effects on the intestinal barrier function and modulate immunity (Praveschotinunt et al., 2019).

Under colitis conditions, sulphate-reducing bacteria generate hydrogen sulfide, which is converted to thiosulfate by host enzymes. Based on a new finding in marine microbiology, a bacterium called *Shewanella halifaxensis* HAWEB4 has been used to detect thiosulfate, an inflammation biomarker. Additionally, *E. coli* has been used to clone and optimize the corresponding genes, resulting in a greater dynamic range of ligand activation by combining various promoters and ribosome binding sites (Daeffler et al., 2017).

The engineered bacteria have also been designed for oral delivery. It causes RNA interference and mediates therapeutics to reduce signs and symptoms of inflammatory bowel disease. The genetically stable and non-infectious *E. coli* MDS42 strain was developed to produce invasin and listeriolysin O cytolysin continuously. Such invasive proteins first target the epithelium and then degrade in the phagosome. This process permits the plasmid delivery encoded with small hairpin RNA (shRNA) in the cytoplasm of target cells capable of targeting tumor necrosis factor (TNF). Such steps cause a significant reduction of TNF and other cytokines and meanwhile also reduce the inflammation in DSS-induced colitis. This strategy is safe with no side effects so it could be used as a modern approach to target TNF and other inflammatory mediators (Ferenczi et al., 2021). Recently, An et al. (2020) designed a living glue system by inspiring the quality of self-regeneration of organisms. This system provides biomedical applications with the ability to sense heme coordination complexes and then respond by repairing the leakage sites within a microfluidic device channel. It could be used in the future to treat gastrointestinal bleeding (An et al., 2020).

### Infections

**FIGURE 6:**
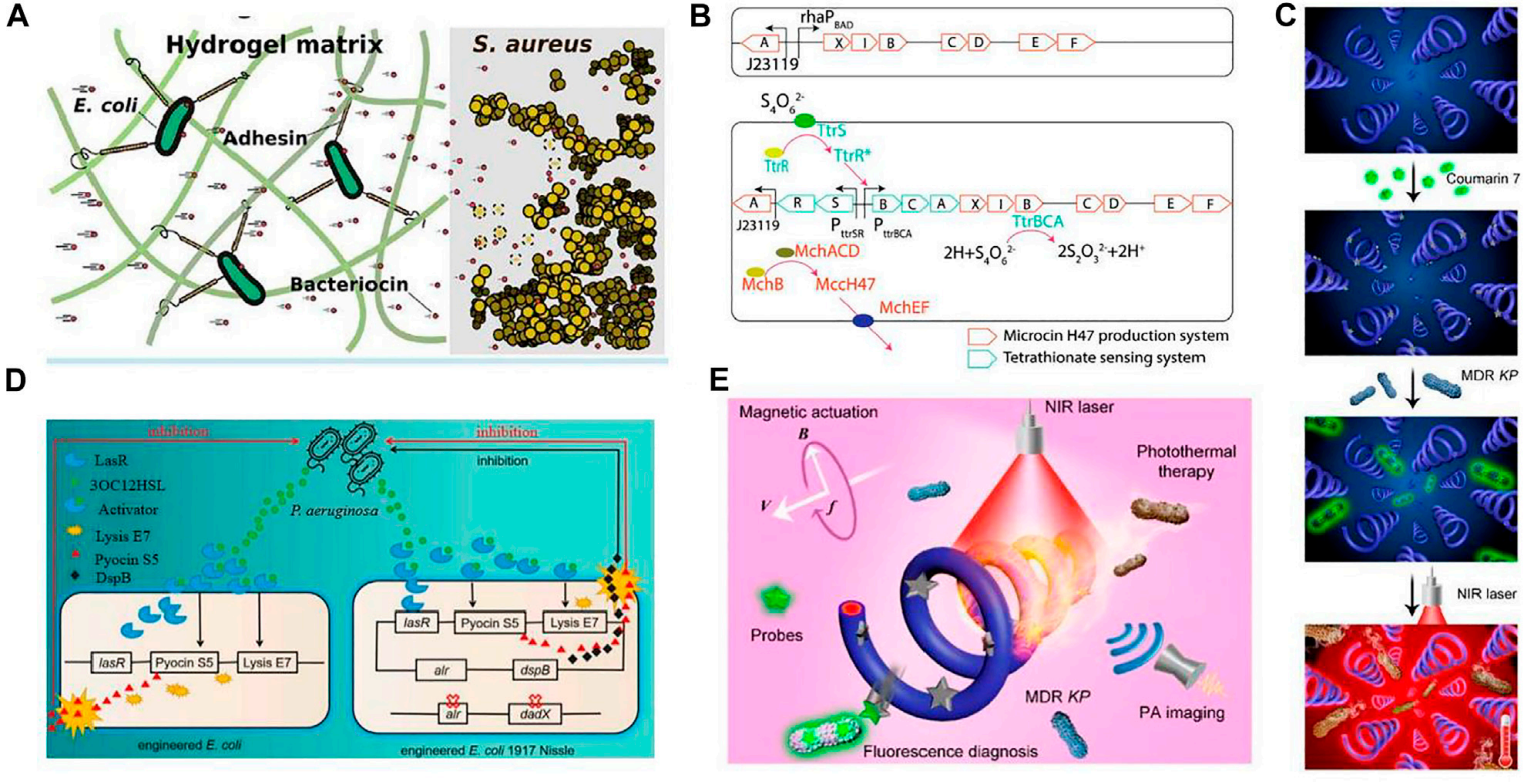
Applications of engineered bacteria for treating infection. **(A)** A novel ELM 'PATCH' has been designed: The engineered *E. coli* release antimicrobial enzymes in a hydrogel material to target *S. aureus*. Reproduced with permission from Guo et al. (2020), Copyright 2020, American Chemical Society. **(B)** The engineered EcN consists of a plasmid system carrying mchAXIBCDEF and ttrRSBCA, producing MccH47 in the presence of tetrathionate. As a result, inhibit and compete with the growth of Salmonella. Reproduced with permission from Palmer et al. (2018), Copyright 2018, American Chemical Society. **(C)** *In vitro* testing of fluorescence (on-off) for bacterial detection and photothermal antibacterial efficiency. Schematic illustration comprises a coumarin 7 probe, fluorescence detection, and photothermal treatment. Reproduced with permission from Xie et al. (2020), Copyright 2020, American Chemical Society. **(D)** A quorum-sensing molecule, 3OC12HSL released by *P. aeruginosa* binds with LasR to form a complex. This complex LasR-3OC12HSL enhances E7 lysis protein and S5 pyocin production. The right section is the modified form of this system. The removal of alr and dadX genes in *E. coli* enhances the stability of the plasmid having the alr gene. Another difference is the addition of dspB, expressing anti-biofilm enzymes Reproduced with permission from Yang et al. (2021), Copyright 2021, Frontiers. **(E)** Schematic diagram depicted photoacoustic (PA) imaging, fluorescence diagnosis, and photothermal treatment of Klebsiella pneumonia infection by engineered spirulina (PDA-MSP). The straight and circular arrows are highlighted for the translational direction of the PDA-MSP and rotational direction of the applied magnetic field, respectively. The symbols B and f represent the strength and frequency of the magnetic field while V is the translational velocity of the PDA-MSP. Reproduced with permission from Xie et al. (2020), Copyright 2020, American Chemical Society.

The latest studies reported the antimicrobial activities of engineered microbes, as shown in Figure 6. Antimicrobial activities with the integration of hydrogels have gained interest in the biomedical field because they provide high capacity and ease in the release of drug loading. A novel engineered living material, ”Platform for Adhesin-mediated Trapping of Cells in Hydrogels” (PATCH) was designed. On the application of PATCH, the engineered *E. coli* releases antimicrobial enzymes inside the hydrogel material, targeting *Staphylococcus aureus* and reducing the chances of increasing antibiotic resistance. Because of the rising issues in resistance strains, Methicillin Resistance *Staphylococcus aureus* (MRSA) is difficult to handle using antibiotics. So, the pentaglycine bridges in the cell wall of *Staphylococcus aureus* can be targeted by lysostaphin. Hence, it offers a treatment strategy against antibiotic-resistant infections (Guo et al., 2020).

Similarly, *E. coli* has been engineered to treat *Salmonella* infection, which causes inflammation in the gut. The engineered strain consists of a plasmid-based system that can sense and utilize tetrathionate, an inflammation biomarker. Upon detection, it can also produce the therapeutic substance microcin H47. Hence, the engineered strain was found to positively reduce *Salmonella* infection (Palmer et al., 2018). However, biologically engineered probiotics were created to detect particular metabolites generated by infections and respond accordingly (Yang et al., 2021). For example, *Lactococcus lactis* was designed to sense quorum-sensing signals specifically related to the diarrhea-associated pathogen *Vibrio cholera* (Mao et al., 2018). Such ELM-based therapy is much more effective than traditional therapy, with fewer side effects.

Small scale robotics are considerably used as tetherless to perform their activity in difficult reach sites with less invasive interference. A strategy was proposed in which polydopamine (PDA) was coated on engineered bacteria *Spirulina.* The coating of PDA increases the photoacoustic (PA) magnetized *Spirulina* (MSP), provides characteristics like natural fluorescence and robust propulsion to microswimmers. Moreover, the quenching and diverse reactivity of the surface of PDA’s innate fluorescence allows on and off diagnosis with a fluorescent probe, coumarin. This theranostic potential of microswimmer PDA-MSP can be applied to treat bacterial infection. This antibacterial microswimmer suggests a feasible and functionalized strategy for *in vivo* development against pathogenic bacterial infection (Xie et al., 2020).

No doubt, synthetic biology has a significant role in engineering microbes which has challenged the design of living materials to function as theragnostic. Recently, *E. coli* has been engineered with a toggle switch as a representative tool to evaluate the replicative states in a rat model with chronic infection. During *in vivo* study, it has been examined that the number of was replicative bacteria was usually remained high throughout the state of chronic infection and also was increased in the presence of antibiotics. Unlike *in vitro* experiments, the non-dividing bacteria *in vivo* do not cause antibiotic-resistant infections. So the engineered bacteria can also be used to examine the behaviour of pathogens (Certain et al., 2017; Hwang et al., 2017).

## Challenges and Future Directions

**FIGURE 7:**
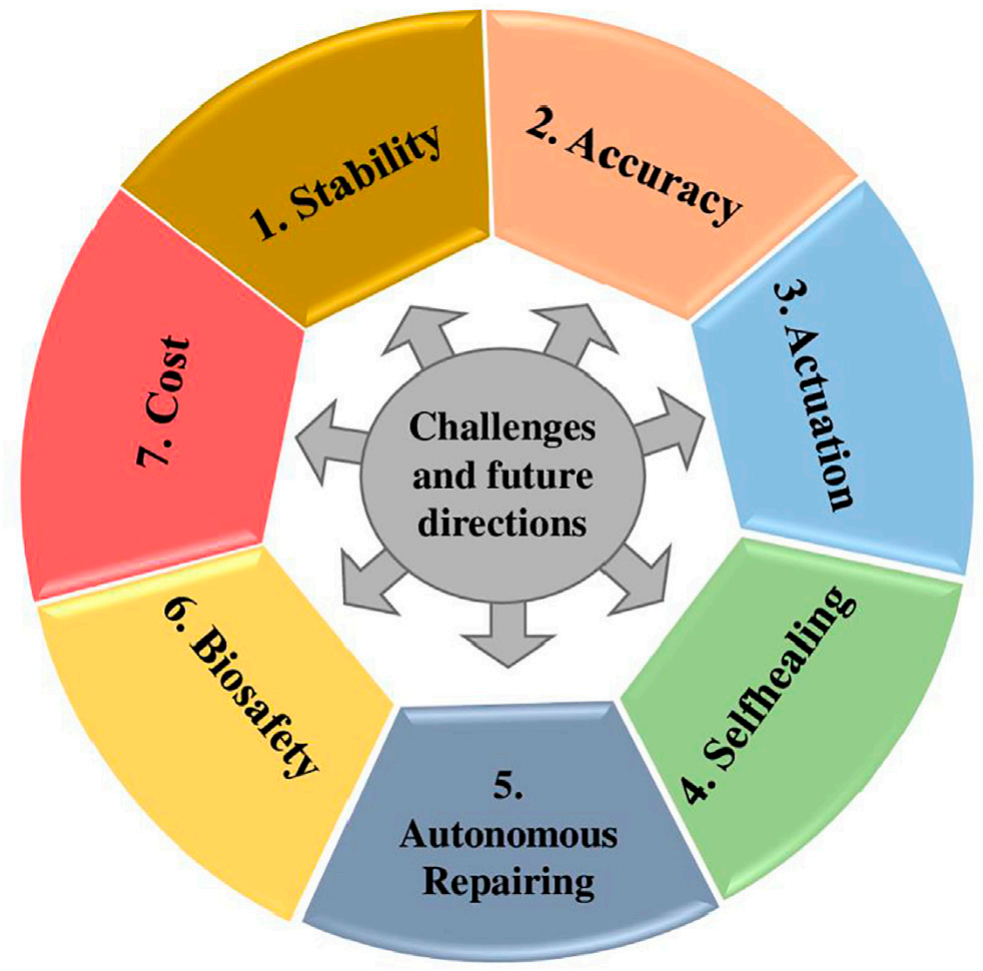
Challenges and future directions.

The combined field of synthetic biology and materials science has a promising effect in creating ELMs with multiple varieties of chemical compositions and hierarchical structures. However, some attributes are difficult to attain in synthetic materials, such as the capacity for self-power, bio-signal responsiveness, and self-sustainability. The application of ELMs for sensing and actuation can bring about dynamic changes by providing new techniques to improve the sensitivity and biocompatibility of the device, allowing easier integration with biological systems (Appiah et al., 2019). In contrast, there are also some issues and challenges as shown in Figure 7 that should be addressed to attain these goals.

### Stability

Implanting genetically modified cells onto a matrix has a short life without a renewable supply of nutrients and metabolic waste management. Consequently, extending the lifetime of the viable cells within the sensors is a critical issue for the sensing power of ELM-based devices. The utilization of bacterial spores is a viable option (González et al., 2020). But for cell sustainability, a more systemic system for integration of various channels and compartments in ELMs based devices would be required. While the other promising solution is the co-culturing and assembly of various coordinating cells.

### Accuracy

ELM-based sensors with quantitative and accurate signal detection still depend on wired connections, therefore limiting their applicability in areas where portable devices are required. Rapid advancements in micro-electromechanical systems (MEMS) and nano-electromechanical systems (NEMS) have enabled the creation of miniature data collection and processing devices. The combination of ELMs with micro and nano-electromechanical devices will offer the potential to build fully functioning and miniaturized ELM-based sensors.

### Actuation

The majority of current research on ELM-based actuators is limited to basic contractile motion or physiochemical bending. This may be accomplished by creating hierarchical 3D structures at the biochemical level, in which cells are placed in predetermined places with varying orientations. To achieve this goal, efficient patterning and alignment with high precision of the extracellular matrix would be required.

### Selfhealing

It is critical to create and integrate living entities with synthetic self-healing and biocompatible materials to accomplish the entire self-healing structure. In recent years, there has been vast and rapid progress in the use of various techniques, such as reformation and reshuffling of covalent bonds, diffusion, flow, shape-memory effects, and combinations thereof, all of which have been used to create self-healing polymeric materials (Wang and Urban, 2020).

### Autonomous Repairing

ELMs for autonomous repairing lack adhesive strength and secreting glue rate is considerably slow, thus cannot contribute in those medical applications where robust recovery and high surface attachment is required. Different approaches like computational design, machine-learning, and directed evolution can overcome this limitation by providing robust sensing and strong adhesive modules. Moreover, stability and continuous performance can be achieved by introducing controller modules that maintain the symbiotic relationship.

### Biosafety

In terms of biosafety, the biocontainment systems are beneficial because of couple sensing with circuit-based control kill switches that hinder the spread of genetically engineered bacteria to the surroundings. For this purpose, “essentializer” and “cryodeath” synthetic circuits in *E. coli* has been installed to act as kill switches (Stirling et al., 2020). Moreover, cancer computers have been designed which shows rapid response and superior safety (Han et al., 2020).

### Cost

Finally, ELM-based devices may be very expensive, particularly if genetic editing is involved. Finding commercially viable applications require a fabrication process by developing academia-industry cooperation.

## Conclusion

ELMs are an emerging interdisciplinary class with foundations laid by the joint efforts of synthetic biologists and materials scientists. This class can provide new research avenues for synthetic biology, materials engineering, and nanotechnology. Engineering techniques help to reprogram living entities, such as microbes, as sensing machines that can sense external stimuli and respond to various cell tasks. By doing so, remarkable attributes can be attained, such as high sensitivity and specificity for poor or weak stimuli, robustness, and continuous sensing. Thus, these biological cells or tissues impart living properties to materials, such as bio-sensing, self-regeneration, and molecular computing capabilities. In comparison, material encapsulation provides applications, such as protection, mechanical enhancement, and communication to engineered biological entities. Hence, these genetically engineered microbes shed new light on biodiagnostics and biotherapeutics to address complicated diseases, such as cancers, IBDs, and various infections. We hope that this emerging field will provide a milestone for the personalized medicine era safely and non-invasive. Although this field is growing rapidly, some challenges remain for exploring novel functional materials and developing suitable therapies for clinical trials.
